# Supplementary material for: Identification and Validation of Nutrient State-Dependent Serum Protein Mediators of Human CD4+ T Cell Responsiveness
Source: Nutrients. 2021 Apr 28;13(5):1492. doi: 10.3390/nu13051492 (PMC8146063; doi:10.3390/nu13051492)
Supplement: Supplementary file 1 [file nutrients-13-01492-s001.zip › Suppl Fig4-Re.pdf.zip!/Suppl Fig4-Re.pdf]

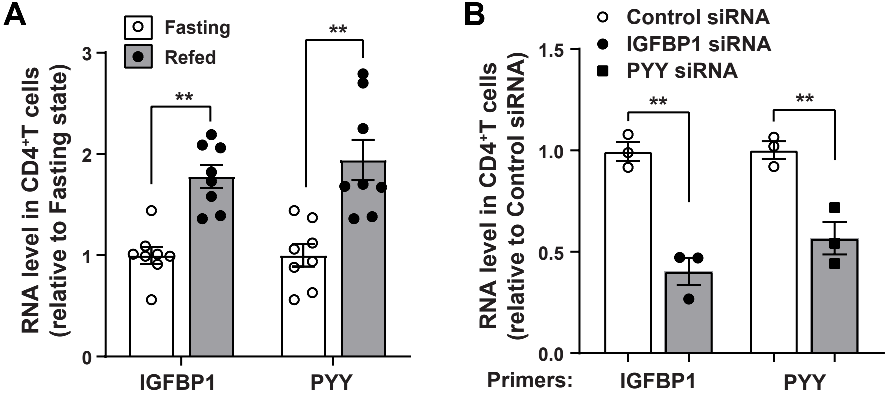

Suppl. Figure S4
